# Breast Reconstruction after Mastectomy

**DOI:** 10.3389/fsurg.2015.00071

**Published:** 2016-01-19

**Authors:** Daniel Schmauss, Hans-Günther Machens, Yves Harder

**Affiliations:** ^1^Department of Plastic Surgery and Hand Surgery, Klinikum rechts der Isar, Technische Universität München, Munich, Germany; ^2^Department of Plastic, Reconstructive and Aesthetic Surgery, Ospedale Regionale di Lugano, Ente Ospedaliero Cantonale, Lugano, Switzerland

**Keywords:** breast reconstruction, breast cancer, mastectomy, DIEP flap, breast implants, autologous fat grafting

## Abstract

Breast cancer is the leading cause of cancer death in women worldwide. Its surgical approach has become less and less mutilating in the last decades. However, the overall number of breast reconstructions has significantly increased lately. Nowadays, breast reconstruction should be individualized at its best, first of all taking into consideration not only the oncological aspects of the tumor, neo-/adjuvant treatment, and genetic predisposition, but also its timing (immediate versus delayed breast reconstruction), as well as the patient’s condition and wish. This article gives an overview over the various possibilities of breast reconstruction, including implant- and expander-based reconstruction, flap-based reconstruction (vascularized autologous tissue), the combination of implant and flap, reconstruction using non-vascularized autologous fat, as well as refinement surgery after breast reconstruction.

## Introduction

Breast cancer is the leading cause of cancer death among women worldwide with ~1.7 million new diagnoses and 521.900 deaths in 2012 (1). One important modality of breast cancer therapy is surgical treatment, which has become increasingly less mutilating over the last century.

William Halsted introduced radical mastectomy including resection of the breast and its underlying pectoralis major muscle in order to cure all stages of breast cancer at the end of the nineteenth century (2). Approximately 40 years later, Patey described a less radical modified type of mastectomy with preservation of the pectoralis major muscle yielding comparable local control and overall survival compared to Halsted (3). In 1985, Fisher et al. introduced the concept of breast conserving therapy (BCT), demonstrating that lumpectomy – by that time regarded as segmental mastectomy – followed by adjuvant radiotherapy of the remnant breast in patients with stage I and II breast cancer was indeed associated with an increased local recurrence rate, yet resulted in equal survival rates compared to mastectomy (4). Oncoplastic breast surgery, i.e., reshaping of the breast after local tumor resection, has shown to allow larger tumor excision, yet conserving large parts of the breast, maintaining shape (5) and resulting in improved quality of life and self-esteem (6). While surgical breast cancer treatment decreased in radicalness and invasiveness, breast cancer guidelines were defined, breast cancer screening programs were initiated, and breast centers offering an interdisciplinary and comprehensive therapeutical approach for breast cancer were established. This resulted in an increased detection and treatment of predominantly early breast cancers with improved survival rates and consequently superior esthetic outcome. Nowadays, BCT is a safe treatment for most women with early-stage breast cancers and can be safely applied in 70–80% of the cases requiring surgical tumor removal (7). Though, the primary goal of BCT is to preserve shape and, to a lesser extent, size of the breast in order to best match the contralateral breast. Thereby, one should take into account that postoperative radiotherapy may result in some extent of tissue shrinkage (8). Although decreasing in number over the last two decades, the rate of mastectomy has again increased lately due to the detection of multifocal tumors, tumors with an extended *in situ* proportion that is difficult to delimit and due to an unfavorable breast-to-tumor size ratio in rather thin patients with small-to-intermediate sized breasts. Furthermore, the awareness of the disease itself in the female population and the relatively frequent detection of a genetic predisposition to breast cancer (i.e., BRCA-1, BRCA-2, p53) have confirmed this trend toward an increased rate of mastectomy, be it curative or prophylactic (9).

Although BCT remains the absolute gold standard for surgical breast cancer treatment, many women must or wish to undergo mastectomy. Consequently, reconstruction of the breast must be offered, particularly in young patients. This article provides an overview of various reconstruction techniques of the female breast after both, breast cancer-related and prophylactic mastectomy. This article does not cover partial breast reconstruction after extensive breast conservative therapy.

## Mastectomy

**Figure 1:**
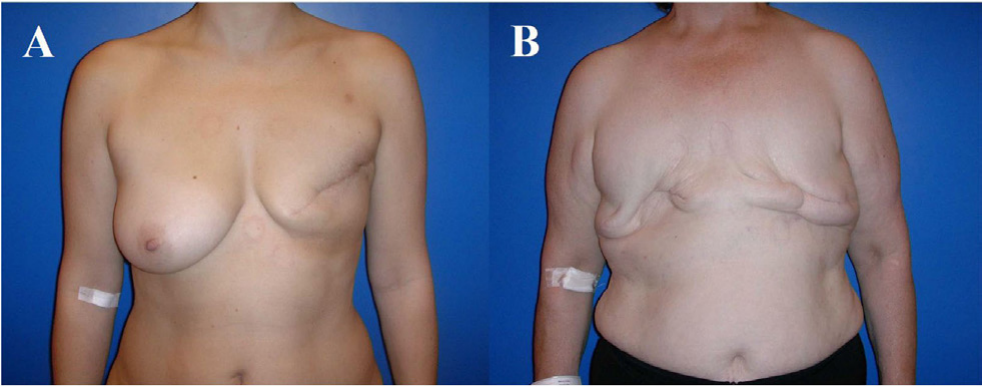
**The 43- and 63-year-old patients after modified radical mastectomy of the left breast (A), respectively, of both breasts (B)**. Indication for autologous reconstruction with a microvascular flap, particularly if skin and fat excess is available and adjuvant radiotherapy has been performed.

Mastectomy aims at resecting as much breast tissue as possible, knowing that glandular tissue will almost always remain in the region of the inframammary fold (10). Nowadays, basically two ways of mastectomy are performed, including skin-sparing mastectomy and total ablation of the breast. The latter consists of complete removal of both, breast skin and glandular breast tissue (Figure 1), whereas skin-sparing mastectomy preserves as much of the breast’s skin envelope as possible, including the areola and the nipple (skin-sparing mastectomy, areola-sparing mastectomy, nipple-sparing mastectomy, skin-reducing mastectomy) and the inframammary fold. Furthermore, biopsy scars and skin overlying a tumor or even infiltrated by the tumor are excised in order to reduce the risk of local recurrence (11). Provided that the oncological indication is correct, skin-sparing mastectomy has been associated with equal oncological local safety and improved esthetic outcome compared to modified radical mastectomy (10). Furthermore, the need for secondary surgery to adjust the contralateral breast in order to achieve symmetry is reduced after skin-sparing mastectomy, particularly if autologous reconstruction with flaps is used (12).

Lately, prophylactic bilateral mastectomy has to be offered more and more frequently due to the increased detection of patients carrying a genetic mutation or predisposition to develop breast cancer (e.g., BRCA-1, BRCA-2, p53). Understandably, these women have high demands to the esthetic outcome that can be overcome with nipple-sparing mastectomy being accepted as the gold standard in patients with prophylactic mastectomies (13).

Noteworthy, skin- and nipple-sparing mastectomies are associated with a high rate of ischemia-related wound breakdown and necrosis of up to 54%, which is a consequence of a critically impaired blood supply of the distant areas of the often very thin skin flap. Meanwhile, various approaches have been described to decrease ischemia-associated complications of the mastectomy skin flap, including surgical skin reduction of the mastectomy flap, temporary insertion of an expandable implant, and local application of vasodilators. Interestingly, first clinical data have shown that local heat preconditioning was able to safely and significantly reduce ischemia-related mastectomy skin flap complications in patients with skin-sparing mastectomy and immediate breast reconstruction (14).

## Reconstruction Techniques of the Breast

Breast reconstruction depends primarily on the type of mastectomy and may be classified in various ways, such as reconstruction type and reconstruction time point. The latter includes delayed breast reconstruction (DBR; secondary breast reconstruction) and immediate breast reconstruction during the same surgery (IBR; primary breast reconstruction). IBR has the advantage of reducing the total number of surgical procedures. Since breast reconstruction *per se* represents an additional procedure to mastectomy, the potential surgery-related complications of both mastectomy and reconstruction accumulate due to prolonged surgery time, particularly if mastectomy is performed using a skin reducing or skin-sparing approach (hematoma due to bleeding of the mastectomy flap, ischemic complications of the mastectomy flap, infection, etc.), respectively, reconstruction is performed with implants. This has to be taken into consideration in order not to postpone adjuvant therapy, i.e., foremost chemotherapy, to the disadvantage of the patient (15). Despite very effective diagnostic work-up of breast cancer and highly standardized neo- and adjuvant treatment regimes, IBR bears the risk that unforeseen adjuvant radiotherapy may compromise the final result of the reconstructed breast, such as capsular contracture in implant-based reconstructions, respectively flap shrinkage in autologous reconstructions. Therefore, many surgeons may tend to a DBR when using free (microvascular) flaps in cases of an invasive tumor requiring adjuvant radiotherapy. In order not to lose the skin envelope after skin-sparing mastectomy, one can place a spacer until completion of adjuvant therapy. Nonetheless, IBR is advantageously associated with a reduced recovery time, a better esthetic outcome, an improved quality of life, and, finally, lower surgery- and recovery-related costs (16–18).

Nowadays, the seek for bilateral prophylactic mastectomy particularly in women with a genetic predisposition for breast cancer (e.g. BRCA-1, BRCA-2, p53) increases and accordingly represents an ideal indication for IBR of any type, knowing that neither adjuvant chemotherapy nor adjuvant radiotherapy will be required (19).

Generally, three different approaches of breast reconstruction may be considered: (1) breast reconstruction using implants and skin expanders, (2) breast reconstruction using flaps (vascularized autologous tissue), and (3) breast reconstruction using non-vascularized lipoaspirate autologous fat. In the following, the different approaches will be briefly highlighted, including the advantages and disadvantages of each approach.

### Breast Reconstruction Using Implants and Skin Expanders

The use of implants and skin expanders is not only the oldest way to reconstruct a breast but also the quickest and presumably easiest method of breast reconstruction. Accordingly, implant-based breast reconstruction is by far the most often used technique worldwide (9, 20). The prerequisite for implant-based breast reconstruction is an adequate skin envelope that allows covering the implant that is usually introduced in a submuscular plane detaching the medial insertions of the pectoralis major muscle from the ribs.

#### Implant-Based Breast Reconstruction

Basically, the first “implant”-based breast reconstruction was performed 1895 by Vincenz Czerny, who used a patient’s lipoma from the lumbar region to reconstruct a post-surgical asymmetry after tumor removal (21).

**Figure 2:**
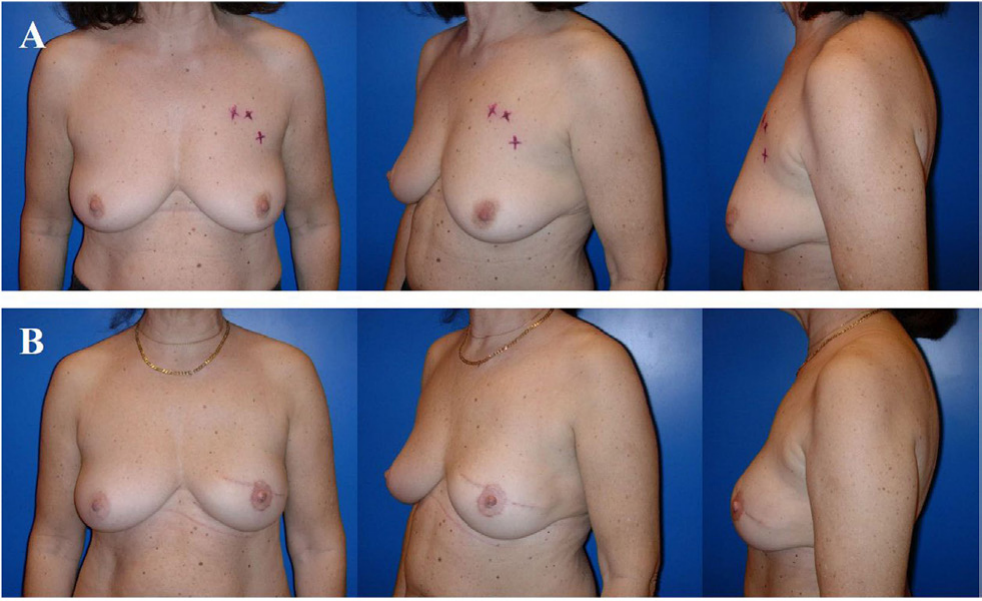
**A 58-year-old patient before skin-sparing mastectomy for multifocal cancer of the left breast (A)**. Four years after primary reconstruction of the left breast using an implant in a subpectoral plane to cover the upper half of the implant and a resorbable mesh to prevent cranialization of the partially detached pectoralis major muscle, as well as reconstruction of the nipple–areolar complex (star flap for the nipple and tatoo of the nipple and neo-areola). Note the almost symmetric size and contour of both breasts **(B)**.

Cronin and Gerow fathered the modern era of silicone gel-filled breast implants and so allowed DBR (22, 23). Nowadays, fifth generation silicone gel-filled breast implants that contain a highly viscous and more or less form-stable gel are usually used. The implants are available in both, round and anatomical shape and vary in width, height, and projection (profile). Implant-based breast reconstruction is used in women who do not want any additional scars (flap harvesting) (Figure 2) or do not have any adequate flap donor site (e.g., lean patient, pre-existing scars, and medical conditions).

**Figure 3:**
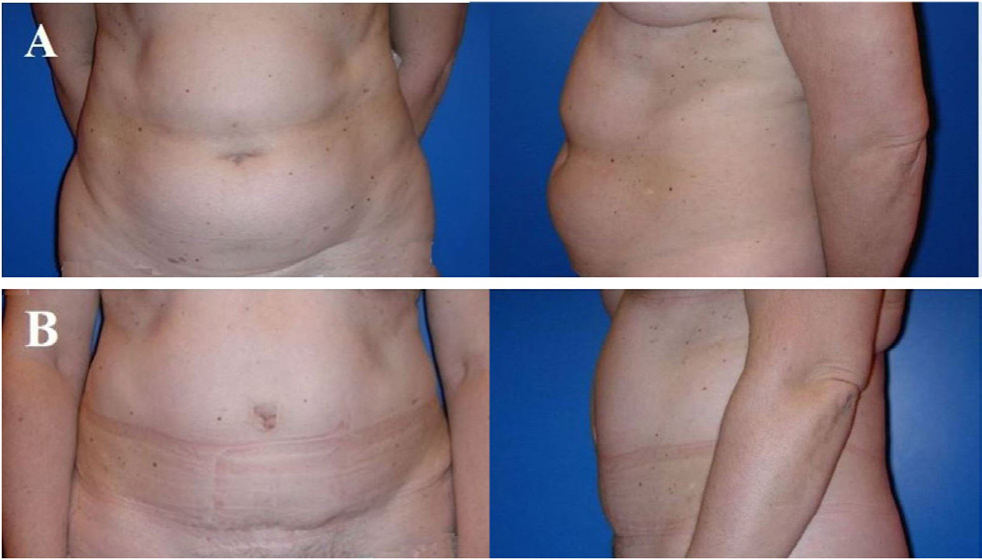
**Typical donor site for abdominal flap-based breast reconstruction**. A 57-year-old patient before **(A)** and 4 years after **(B)** harvesting a microvascular deep inferior epigastric perforator (DIEP) artery flap from the abdominal region. Note the adipocutaneous excess cranially and distally of the umbilicus, the almost invisible scar at the umbilicus and the suprapubic region, as well as the significantly improved abdominal contour [profile view **(A,B)**]. The reconstructed breast of this patient is shown in Figure 6.

Implant-based breast reconstructions prone to develop implant-related local complications during the subsequent 10 years with a risk for a reoperation of 70% (24). Approximately 25 and 35% of the patients are being diagnosed with severe capsular contracture and, respectively, implant rupture (25). This high complication rate results from the thin skin envelope remaining after mastectomy, which does not provide any robust coverage of the implant. This complication rate does neither consider breast shape deformity and asymmetry in the context of mild to moderate capsular contracture nor does it consider an even worse outcome in implant-based breast reconstruction with irradiated skin. *De facto*, breast reconstruction using implants may yield very nice long-term results that suffice many patients, yet the implant will always remain more or less fixed to the thoracic wall and consequently the breast maintains a unique shape, independently from the patient’s posture. Finally, implant-based breast reconstruction will not allow recreating a naturally shaped ptotic breast in most patients, and therefore often requires adaptive surgery of the contralateral breast to achieve symmetry. Though, implant-based breast reconstruction prevents from “collateral damage,” such as scars, contour deformity, and muscular weakness, as it might be seen after flap harvesting for flap-based breast reconstruction (Figure 3).

#### Expander-Based Breast Reconstruction

The technique of tissue expansion has first been described by Radovan in 1976, and since then, it has been used on a regular base in order to recreate the amount of lost skin after mastectomy through stepwise expansion of the remaining chest skin (26). Ideally, the contralateral breast volume should not exceed a volume of 300–400 g. Typically, patients with pre-expansion of the breast skin undergo secondary breast reconstruction with implants. In selected cases, especially in young and skinny patients with insufficient skin laxity, yet enough adipose tissue to reconstruct a breast mound of ~300–350 g, one might pre-expand the breast skin after mastectomy in order to substitute the expander with an autologous flap.

The main drawback of skin expansion are the frequent out-patient visits to gradually fill the expander, the need for an additional procedure (i.e., expander removal for permanent implant or flap) and the relatively high rate of complications, such as infection, capsular contracture, and skin perforation (27).

Alternatively, skin expanders can be used as “spacers” after skin-sparing mastectomy in order not to lose the skin pocket. This approach is particularly helpful in patients who are sure to get adjuvant radiotherapy of the skin and/or the thoracic wall. Indeed, postoperative radiotherapy will not significantly increase the rate of flap-related complications (28). Yet, IBR is more and more frequently performed using microvascular (free) flaps despite postoperative radiotherapy. However, we currently do not know at what extent the flap will indurate and shrink at long-term follow-up.

#### Breast Reconstruction with Acellular Dermis

The use of acellular dermal matrices in implant- and expander-based breast reconstruction has lately become more and more popular. Matrices are usually of human, porcine, or bovine origin. They have shown to improve esthetic outcome and reduce implant-related morbidity (29), such as a decreased rate of capsular contracture (30–32), an improved tolerance to radiotherapy, and a more natural anatomical reconstruction of the inframammary fold and final breast contour (33).

Nahabedian demonstrated a high safety and excellent results using acellular dermis in a 12-year follow-up, even in the setting of reconstruction after infection or radiotherapy. However, other authors reported several matrix-related complications, such as hematoma, infection, and foremost late seromas (29). The use of matrices is again and again associated with a rather high rate of early complications. Lardi et al. have demonstrated that these complications were mostly related to patient characteristics and a learning curve, highlighting the importance of patient selection and technical principles (34).

### Breast Reconstruction Using Flaps (Vascularized Autologous Tissue)

The myocutaneous flaps that are being used for breast reconstruction have a long history, although the techniques of today are much more sophisticated than those of the past. Louis Ombredanne from France was the first to use a pedicled pectoralis muscle flap for IBR in 1906. Differently as his colleagues, Ombredanne was the first who wittingly tried to reconstruct not only the skin defect after mastectomy but also the breast mound that was considered at that time a “luxury” procedure with limited indications (35). Almost simultaneously, Tanzani from Italy used the pedicled myocutaneous latissimus dorsi flap to close mastectomy defects for the first time.

Flap surgery for breast reconstruction has been performed on a regular base, since the mid 70s, initially using both, tubed flaps from the abdomen (36) and thoraco-epigastric, necessitating several surgical stages (23). The initial attempts were still not able to really reconstruct the breast mound and therefore primarily aimed at resurfacing the thoracic wall’s defects after radical mastectomy. Finally, it was the introduction of the myocutaneous latissimus dorsi flap with its overlying skin island, as described by Tanzani 70 years earlier, which allowed to restore mastectomy-induced skin loss and to a lesser extent also volume loss (37–39).

Almost at the same time, Bostwick described the combined use of the myocutaneous latissimus dorsi flap and a silicone implant to consistently provide adequate skin coverage, respectively, to restore the breast mound in postmastectomy reconstruction (40).

**Figure 4:**
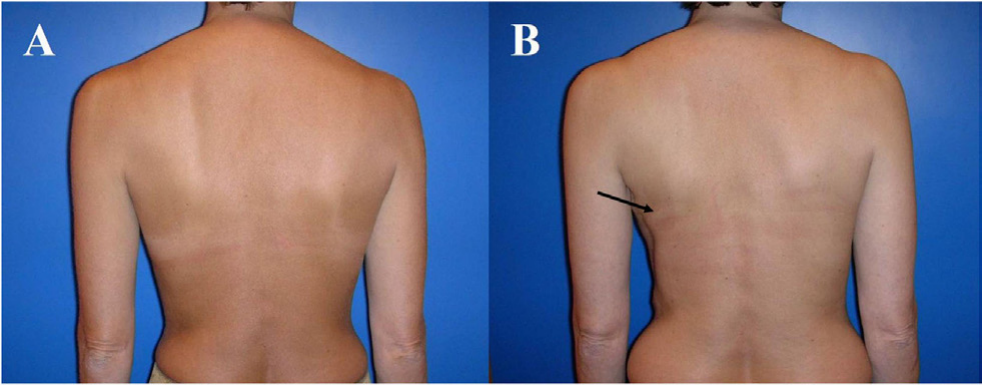
**Typical donor site for myocutaneous latissimus dorsi flap-based breast reconstruction**. A 36-year-old patient before **(A)** and 2 years after secondary reconstruction of the left breast using a pedicled myocutaneous latissimus dorsi flap **(B)**. The skin island is harvested along the posterior axillary line. Note the well concealed scar (usually in the bra-line) that does not interfere with the back of the patient, yet skin and muscle harvesting result in a slight contour deformity of the periscapular region [arrow; **(B)**]. The reconstructed breast of this patient is shown in Figure 5.

**Figure 5:**
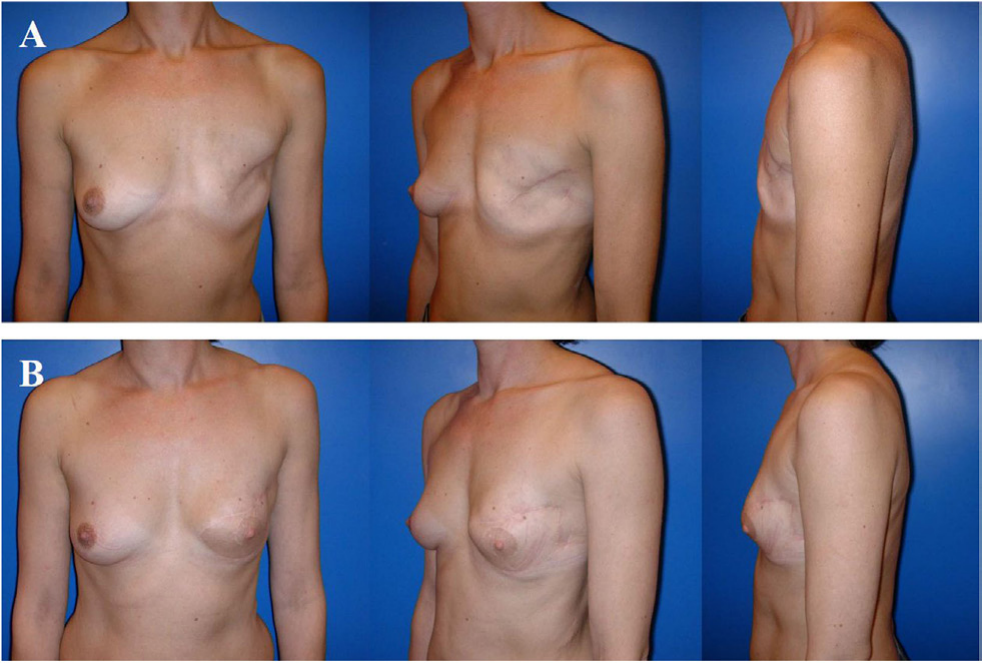
**A 36-year-old patient 3 years after modified radical mastectomy of the left breast and adjuvant radio-chemotherapy**. Note the oblique scar and rather large skin envelope in a thin patient **(A)**. Two years after secondary reconstruction of the left breast using a pedicled myocutaneous latissimus dorsi flap without implant and reconstruction of the nipple–areolar complex (star flap for the nipple and tatoo of the nipple and neo-areola). Note the almost symmetric neckline and the slight volume loss of the lower pole of the breast resulting in contour deformity **(B)**. The donor site of this patient is shown in Figure 4.

The advantage of the latissimus dorsi flap is its rather consistent anatomy and therefore easy flap harvest. However, flap transfer from the back can be associated with highly visible scars, contour deformity of the thorax ventrally and the back dorsally as well as animation of the skin/muscle-implant complex of the pectoralis major respectively latissimus dorsi muscle due to innervation of the latter one (Figure 4). Otherwise, the muscle undergoes atrophy of 50–75% of its volume unconditionally, almost always requiring an implant to restore volume, unless the patient is rather thin (Figure 5).

Since this reconstructive approach combines two basic techniques of reconstructive surgery, i.e., skin replacement with the flap and volume restoration with the implant, the patients are subject to an accumulation of the two technique’s morbidities, which might be significant, particularly years after reconstruction. Tarantino et al. demonstrated that 57% of the patients treated with a latissimus dorsi flap and implants had revisional surgery for implant replacement or implant removal after a mean follow-up of 10 years, and concluded that the indication for this procedure should be restricted to patients who do not qualify for either implant-based or flap-based breast reconstruction (41).

In 1987, Hokin and Silfverskiold described the use of an extended latissimus dorsi flap to avoid the use of an implant. The flap’s volume was significantly increased by dissecting the subcutaneous fat surrounding the skin island (42). Unfortunately, donor site morbidity increased dramatically, including prolonged seroma respectively wound dehiscence rate, and contour deformity (43).

The true progress in flap-based breast reconstruction occurred in 1982 when Hartrampf and colleagues used the cranially pedicled rectus abdominis muscle flap with a horizontally oriented adipocutaneous skin island (TRAM flap) supplied by the deep superior epigastric artery to anatomically reconstruct volume and shape of the breast in one single stage without using implants (44).

Although this procedure was able to both, restore the ablated breast and improve abdominal contour despite scars at the umbilicus and the waistline, following significant disadvantages have to be taken into consideration: a high tissue-to-blood supply ratio of the flap, protracted recovery of the patient and abdominal wall weakness, including bulging and herniation due to sacrifice of the rectus abdominis muscle and large part of its anterior fascia.

**Figure 6:**
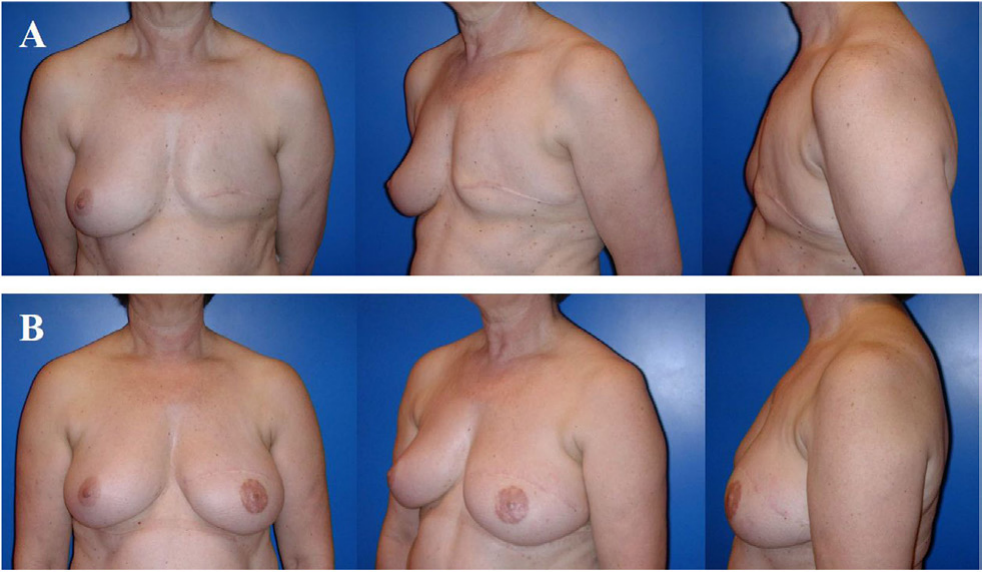
**A 57-year-old patient 2 years after modified radical mastectomy of the left breast and adjuvant radio-chemotherapy**. Note the lack of skin and volume **(A)**. Four years after secondary reconstruction of the left breast using a microvascular deep inferior epigastric perforator (DIEP) artery flap from the abdominal region and reconstruction of the nipple–areolar complex (star flap for the nipple and tatoo of the nipple and neo-areola). Note the almost symmetric size and contour of both breasts without corrective surgery of the non operated contralateral breast **(B)**. The donor site of this patient is shown in Figure 3.

To overcome these drawbacks of the donor site of the pedicled TRAM flap, Arnez and colleagues and Grotting et al. popularized the free TRAM flap, i.e., the microvascular anastomosis of at least one artery and one vein of the flap to recipient vessels. In doing so, the authors were able to demonstrate a more limited harvest of the rectus abdominis muscle, a safer transfer due to improved perfusion originating from the larger caudal pedicle (deep inferior epigastric artery instead of deep superior epigastric artery), and an improved medial breast contour due to the lack of tunneling of the flap’s cranial pedicle (45, 46). Further refinement of the surgical technique over time aimed at decreasing as much as possible the weakening of the abdominal wall despite transferring most of the abdominal skin and its underlying subcutaneous tissue, including muscle sparing free TRAM flap (47), fascia sparing free TRAM flap (48), to finally achieve complete muscle preservation. The latter was obtained by dissecting the vascular pedicle of the adipocutaneous abdominal flap perforating the rectus abdominis muscle (deep inferior epigastric perforator (DIEP) artery flap) as described by Allen and Treece (49) and Blondeel and Boeckx (50) (Figure 6). The concept of this so-called “perforator flap” or DIEP flap has somehow revolutionized breast reconstruction by maximizing the amount of safe tissue transfer, yet minimizing donor site morbidity. Abdominal tissue is very suitable for breast reconstruction, since many patients have a certain abdominal excess of skin and fat. Consequently, autologous breast reconstruction using a DIEP flap nowadays represents the gold standard. In case of concomitant chronic lymphedema of the arm after sentinel lymph node biopsy, axillary lymph node dissection and or/radiotherapy of the lymph node basins, one can surgically address this problem using lymphaticovenous anastomosis or microvascular lymph node transfer. The latter can easily be combined with a DIEP flap, since the flap mostly consists of the lymph nodes in the groin area lateral to the femoral vessels and depends on the pedicle originating from the superficial inferior epigastric vessels (51). Given that not every women is suitable for breast reconstruction using abdominal skin and fat, many more donor sites were described in the following years, aiming at harvesting the most suitable microvascular flap to best personalize breast reconstruction. This included, among others, the superior gluteal artery perforator (sGAP) flap (52), the inferior gluteal artery perforator (iGAP) flap (53) from the gluteal region, the fasciocutaneous infragluteal (FCI) flap (54), the profunda femoral artery perforator (PAP) flap (55) from the infragluteal region, and the transverse myocutaneous gracilis (TMG) flap from the inner thigh region (56).

Nowadays, the internal mammary artery and its concomitant vein are predominantly used as the recipient vessels. Alternatively, the arterial branches originating from the subscapular artery (e.g., thoracodorsal artery, circumflex scapulae artery) or sternal perforators arising through the pectoral muscle are used.

Although breast reconstruction using autologous flap tissue allows a natural and durable result, flap harvest will cause a “collateral damage” at the flap’s donor site, including potential surgery-related complications, scars, contour deformity, and functional impairment. Furthermore, microvascular flap-based breast reconstruction is not only technically more demanding, but also requires more infrastructures within a breast reconstruction unit, as compared to implant-based breast reconstruction (Table 1).

**Table 1:** **Advantages and disadvantages of implant-based versus autologous tissue-based techniques of breast reconstruction**.

	Implant-based breast reconstruction	Autologous tissue-based breast reconstruction
Duration of surgery (h)	1–2	4–6
Infrastructural effort	Low	High^a^
Surgical complexity	Low	High
Donor site	None	Depending on flap^b^ (abdominal, thigh, gluteal, dorsal region)
Complication rate (30 days) (implant-, respectively, flap-related) (%)	2–4	2–4
Complication rate (long-term)	Higher (due to capsular contracture)	Lower
Long-term reoperation rate	More likely	Less likely
Patient satisfaction	Short-term	Long-term

*^a^Microscope, specific instruments, trained personnel (nurses in OR)*.

*^b^Discomfort, pain, scars, abdominal bulging, hernia, asymmetry, and contour deformity*.

### Fat Graft-Based Breast Reconstruction Using Non-Vascularized Lipoaspirate Fat

Autologous fat grafting (AFG; lipografting, lipofilling) describes the harvesting of the patient’s fat using liposuction followed by its reinjection into the tissue to be corrected or augmented. Fat grafting to the breast is more than 100 years old since Holländer corrected a retracted scar after mastectomy by injecting parceled fat into the scar (57). AFG to the breast has become a popular tool over the last 20 years, both in esthetic and reconstructive surgery. Regarding the breast, AFG has proven to be particularly effective to correct post-surgical irregularities, such as contour deformities and volume asymmetries after BCT, “rippling” after implant-based reconstruction and improvement of the transition zone between flap and skin in the neckline (58–60), as well as the preparation of the postmastectomy irradiated chest wall prior to implant placement (61). In selected cases, *de novo* reconstruction of the breast by means of AFG has shown very promising results. The patient must have several donor sites equipped with fat, because the reconstructive procedure usually takes four to six stages of fat grafting, each separated by 3 months at least (62). Irradiated skin does almost preclude this approach, since injected fat is not engrafted as desired (63).

Autologous fat grafting is a “natural” filler, and unlike synthetic fillers will neither induce any foreign body reaction nor be resorbed completely. Today, harvesting of the fat is discussed, among others, with regard to composition of the infiltration solution, to diameter and shape of the harvesting cannula and to suction forces. In order to be structural, injection of the fat should be performed in small aliquots using blunt cannulas in multiple directions and multiple layers. This multi-planar approach maximizes the fat-to-tissue contact, thereby the exposition of non-vascularized fat to vascularized host tissue (64). Consensus exists on the fact that fat may not be injected into the glandular tissue of the breast. Commonly, 60–70% of the injected fat is engrafted to the host tissue. Fat necrosis and oil cysts are common complications after AFG and occur in ~5% (65). Unfortunately, necrosis of the grafted fat might also be associated with microcalcifications, which sometimes may be difficult to distinguish from malignant breast cancer-associated microcalcifications (66). Presumably, the radiologist is an expert, fat grafting-induced microcalcifications do no impact on the radiological follow-up (67). Yet, this fact may unsettle the patient who has to appear for regular follow-up imaging and eventually undergo diagnostic biopsy to exclude malignancy.

Currently, fat grafting to the breast is controversially discussed, particularly in the presence of remaining glandular breast tissue, as, for example, after BCT. Grafted fat that naturally contains progenitor and stem cells has lately been associated with breast cancer progression and metastasic spread in an experimental setting (68, 69). Despite the lack of prospective follow-up studies, fat grafting to reconstruct or to refine a breast after mastectomy and/or after breast reconstruction – BCT not included – is nowadays considered safe (70–72).

## Refinement Surgery after Breast Reconstruction

**Figure 7:**
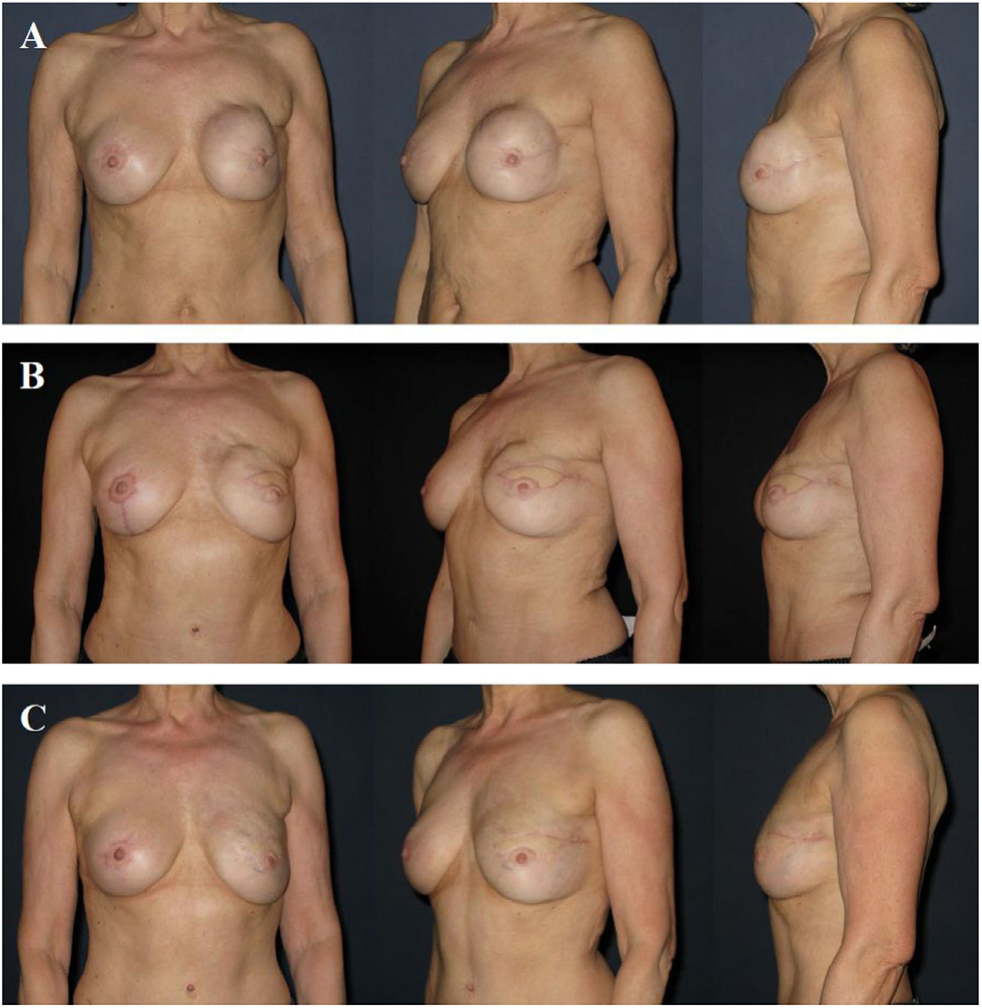
**A 58-year-old patient 2 years after modified radical mastectomy of the left breast, adjuvant radio-chemotherapy, and secondary expander–implant-based reconstruction**. The patient developed a capsular contracture Baker grade IV with a hard, deformed, and painful breast fixed to the thoracic wall **(A)**. One year after implant removal, radical capsulectomy and secondary reconstruction of the left breast using a microvascular deep inferior epigastric perforator (DIEP) artery flap from the abdominal region. Note the contour deformity in the neckline and upper pole region of the breast resulting from partial fat necrosis of the flap **(B)**. Approximately 1.5 years after refinement of the contour deformity using two sessions of autologous fat grafting. Note the almost symmetric size and contour of both breasts **(C)**.

After breast reconstruction, particularly if breast reconstruction is performed unilaterally, refinement surgery may be necessary to reach symmetry of the breasts with regard to shape, contour, and size. The procedures usually consist of mastopexy, breast reduction, or breast augmentation using implants. Nowadays, AFG is often used to correct small volume asymmetries and contour deformities. The latter may occur after implant-based breast reconstruction (e.g., “rippling”), as well as after flap-based breast reconstruction (e.g., partial fat necrosis of the flap, transition zone between flap and neckline cranially). Refinement surgery is usually offered not earlier than 3 months after reconstruction or 6 months after completion of adjuvant radiotherapy. Fat grafting has often to be repeated. Its engraftment rate is ~60% (~40% fat resorption) (Figure 7). Last but not least, mastectomy is associated with the loss of the nipple–areolar complex (except for nipple-sparing mastectomy), requiring its reconstruction. Many techniques of reconstruction are available, including local flaps of the adjacent skin, skin grafts, tattoo, and a combination of all techniques.

## Conclusion

Breast cancer is the leading cause of cancer death in women. Its surgical approach has become less and less mutilating, allowing for 70–80% of the operated cases to undergo (BCT) that has proven to be as safe as mastectomy with regard to overall survival. In other words, 20–30% of the operated women are subjected to mastectomy. Since ~25 years, the skin-sparing mastectomy approach is an alternative to ablation of the breast allowing for better esthetic results due to preservation of the breast’s skin envelope, yet from an oncological point of view as safe as mastectomy. Other than mastectomy, skin-sparing mastectomy needs immediate reconstruction in order not to lose the skin envelope that unreconstructed will inevitably retract and shrink to the level of the thoracic wall. Nowadays, breast reconstruction should be personalized at its best, first of all taking into consideration not only the oncological aspects of the tumor, neo-/adjuvant treatment and genetic predisposition, but also its timing (IBR versus DBR), as well as the patient’s condition and wish. Despite this complex decision-making including many aspects, the overall number of breast reconstruction has lately considerably increased. Breast reconstruction itself can basically be classified into three categories, including (1) implant- and expander-based breast reconstruction, (2) flap-based breast reconstruction (vascularized autologous tissue), a combination of both (flap and implant), and (3) breast reconstruction using fat grafting (non-vascularized autologous lipoaspirate fat). However, fat grafting is predominantly used to refine post-reconstructive asymmetries. Nowadays, it is of importance that every woman having a high risk constellation (family history), being diagnosed with a genetic mutation and/or being affected with breast cancer gets the possibility to be presented to a multidisciplinary board of a certified breast center prior to surgery in order to be informed about all treatment modalities, including the various modalities of breast reconstruction. The goal of this multidisciplinary board is to best personalize breast reconstruction, of course putting to the fore the adequate oncological treatment. The patients also need to know the advantages and disadvantages of any reconstructive option, including the presumably less complex implant-based techniques that may result in high temporary satisfaction without any donor site morbidity and likelihood of reoperation due to capsular contracture and the clearly more complex flap-based techniques that will yield in high long-term satisfaction with the risk of donor site-associated complications.

## Conflict of Interest Statement

The authors declare that the research was conducted in the absence of any commercial or financial relationships that could be construed as a potential conflict of interest.
